# Effects of Passion, Experience, and Cultural Politics on Classical Musicians’ Practice During the COVID-19 Pandemic

**DOI:** 10.3389/fpsyg.2022.888678

**Published:** 2022-05-02

**Authors:** Guadalupe López-Íñiguez, Gary E. McPherson, Francisco J. Zarza Alzugaray, Rolando Angel-Alvarado

**Affiliations:** ^1^Sibelius Academy, University of the Arts Helsinki, Helsinki, Finland; ^2^Melbourne Conservatorium of Music, University of Melbourne, Parkville, VIC, Australia; ^3^Department of Musical, Plastic and Bodily Expression, University of Zaragoza, Zaragoza, Spain; ^4^Music Institute, Alberto Hurtado University, Santiago, Chile

**Keywords:** COVID-19, cross-cultural study, expert, multigroup invariance analysis, music practice, passion, professional musician, self-determination theory

## Abstract

The widespread cancelation of cultural events during the early 2020 stages of the COVID-19 pandemic led professional performing musicians across the world to experience an increasing economic fragility that threatened their health and wellbeing. Within this “new normal,” developing countries have been at a higher risk due to their vulnerable health systems and cultural policies. Even in such difficult times, the music profession requires musicians to keep up their practicing routines, even if they have no professional commitments. This is because high level technical and expressive skills are crucial to sustaining a music career at a high performance level. However, it could be expected that not all musicians might have had the same engagement with music practice during lockdowns. In this study, we studied the experiences of 309 professional classical musicians based in European and Latin American countries with different levels of performing experience to examine their passionate (or lack thereof) engagement with music practice. Through the mixed methods combination of multigroup invariance and narrative analyses, we identified distinct profiles of musicians who displayed more harmonious or more obsessive passion orientations before and at the peak of the pandemic. We observed that musicians with higher levels of harmonious passion in particular were more capable of sustaining their practice at the peak of the pandemic and that these musicians were mostly located in Latin America—a paradox, considering that cultural politics supporting the careers of professional performing musicians and entrepreneurial education in Latin America are lacking to a great extent, especially in comparison with the European context. We explain this in terms of the “forced” self-management embraced by musicians in Latin American countries who want to engage with music practice both before and during the COVID-19 pandemic even if the music profession does not generate enough revenue for them.

## Introduction

Since the outbreak of the COVID-19 pandemic (Coronavirus Disease) toward the end of 2019, business, trade, tourism, and the world economy more generally have experienced dramatic disruptions. Indeed, the pandemic was soon thereafter declared by the World Health Organization as a “Public Health Emergency of International Concern” that, in particular, posed “a high risk to countries with vulnerable health systems” (Sohrabi et al., 2020: 71; WHO, 2020). Within this new reality, the music industry globally—which has been generally characterized by the uncertainty and shifting nature of the music profession before the pandemic (e.g., López-Íñiguez and Bennett, 2020)—has been among the most impacted (e.g., FIM – International Federation of Musicians, European Union, 2020; Hall, 2020), due to the massive cancelation of cultural events and the transition to virtual modes of collaborative and individual music making and teaching/learning.

Among the most serious consequences of political decisions and lockdowns that have impacted the lives of professional musicians are: (1) a severe drop in income and an overall decrease in levels of health and wellbeing (e.g., Schwalje and Hoffman, 2020; Cohen and Ginsborg, 2021; Crosby and McKenzie, 2021; Curry, 2021; Spiro et al., 2021; Vance et al., 2021), (2) motivational difficulties in maintaining music practice routines that are crucial to sustaining professional performing careers (López-Íñiguez et al., 2022), and (3) challenges to provide comprehensive instrumental/vocal learning *via* digital platforms (e.g., Biasutti et al., 2021; Pozo et al., 2022). The sudden and dramatic changes that occurred because of the pandemic resulted in calls for urgent policy agendas to provide basic economic support and resources for musicians, so they could survive until audiences are able to return to concert halls. At the forefront of efforts to advocate for musicians was the need for sensitive leadership and decision making that would ensure musicians maintain a personal sense of professional purpose in times of crises, while at the same time helping to ensure that their performance skills do not deteriorate.

For professional classical musicians—who tend to be passionate about music making (i.e., Bonneville-Roussy and Vallerand, 2020; Vallerand et al., 2022)—the maintenance of artistic skills is typically connected to long hours spent practicing deliberately (Ericsson et al., 1993) and the care and attention required to maintain vocal/instrumental skills across an entire performing career (e.g., McPherson et al., 2019). In many cases however, there is evidence that many musicians lack approaches that might enable them to effectively manage the complex practical and personal challenges that music careers often pose (Gross and Musgrave, 2020).

Considering the above, and despite recent studies outlining the impact of COVID-19 on professional performing musicians’ careers, little is known about the effect that the type of passion displayed by individuals toward their professional music practice. Specifically, we know little about these musicians practice and rehearsing (as compared to performing), or how different years of performing experience influence how they sustain their practicing routines when concert engagements unexpectedly disappear from their agenda and they suddenly face an uncertain future that might end their performance career. In addition, there is also a dearth of information concerning differences between musicians from different geographical regions who belong to diverse socioeconomic, politic, and cultural systems, and how they cope during a period of great uncertainty, such as the COVID-19 pandemic.

## Theoretical Frameworks

### The Dualistic Passion of Professional Musicians: Harmonious vs. Obsessive

Since the 1990s, Robert J. Vallerand and his colleagues have sought to explain a range of issues concerned with passion. This body of psychological research is important because it is among the first attempts to study passion for an activity as compared, for example, to other constructs such as romantic passion (Hatfield and Walster, 1985). Using Self-Determination Theory as the underlying framework, Vallerand et al. (2003) and Vallerand (2008) sought to explain more precisely how the basic psychological needs of autonomy, competence, and relatedness are satisfied as people engage with activities in which they have little control (e.g., school and work) or alternatively choose during their leisure time (e.g., sport and music). Studies were devised to document how individuals show preferences over time for some but not other activities in which they feel empowered or wish to pursue, and how they become passionate about those activities they find most interesting and important to them.

Vallerand (2008) defined passion as “a strong inclination toward a self-defining activity that one likes (or even loves), finds important, and in which one invests time and energy” (p. 1–2). Such activities are self-defining for an individual, an inherent part of their identity, and highly valued and meaningful. Within this conception, there are two types of passion—*obsessive* and *harmonious*—which are distinguished by the degree to which the passionate activity has been internalized within the person’s identity.

*Obsessive passion* “results from a controlled internalization of the activity into one’s identity” (Vallerand, 2008, p. 2) and is typically accompanied by factors external to the person, such as pressure or control from others or an organization that result in feelings of a lack of control. Obsessively passionate musicians, for example, are more likely to feel that others control their professional lives, find it difficult to focus on the activity, feel upset with themselves when experiencing conflict between that activity and other priorities, and experience less positive feelings of affect and flow when engaged in music. They also tend to adhere to a rigid persistence and be less flexible with their involvement in an activity. Most importantly, they tend to experience more frustration and rumination about the activity when they are prevented from engaging with it (Vallerand, 2008).

In contrast, *harmonious passion* results when an activity is autonomously internalized into an individual’s identity (Vallerand, 2008). According to Vallerand (2008, p. 2), “An autonomous internalization occurs when individuals have freely accepted the activity as important for them without any contingencies attached to it.” Feelings of passionate involvement occur because the activity is personally endorsed by individuals who freely choose to do the activity because they do not feel pressured, coerced, or any uncontrollable urge to engage with the activity. Because they can focus on the activity, they are more likely to experience states of flow and positive affect, and when their life situation prevents them from engaging in the activity for which they feel passionate, are able to adapt and focus their attention and energy on other activities that may have a higher priority.

The dualistic explanation of passion seeks to answer the question “How can a person’s life be most worth living?” For Vallerand (2008), “one answer to that question is by having in one’s life a harmonious passion toward a meaningful activity or a cause” (p. 10). The life of professional musicians often involves strict adherence to the instructions of others (e.g., conductors, administration, specified concert rehearsals, and performances) and it is for this reason that this dualistic explanation of passion is one way to differentiate between individuals who typify a profile of obsessive as compared to harmonious feelings when involved with music throughout their professional careers (Bonneville-Roussy and Vallerand, 2020).

### Cross-Cultural Research in Music: Diverse Cultural Politics in Europe and Latin America

The COVID-19 pandemic marks a socioeconomic and cultural milestone in the music industry because, while live events have been postponed or canceled, streaming music services have mitigated the economic damage through recorded music and virtual concerts (Isernia and Lamonica, 2021; Baltà, 2022). So, musicians have had to adapt the working routine to a new multifaceted scenario that demands—in addition to professional skills in music—abilities in production and post-production, management, and even advertising (Barlow and Morrow, 2021). Such new challenges faced by musicians, however, are reliant on a number of geographical contexts. This is because every region enacts cultural policies autonomously (Moreno, 2022). For example, trade of cultural goods and services from developed countries is clearly more efficient than for developing countries (UNESCO, 2022). The pandemic is a global issue, but the effects and impacts of responses to cultural policies vary among nations across the world because each one understands in a particular way their own economic model (see Harari, 2020).

In Europe, the COVID-19 pandemic displayed the economic fragility of cultural and creative sectors in which the workers worked (Baltà, 2022), even though those European industries have received public funds since before the crisis—thus benefiting entrepreneurship, innovation, and growth in various cultural sectors (Dümcke, 2021). During the health crisis, member states from the European Union have supported musicians through structural funds provided by The Coronavirus Response Investment Initiative, which was enacted in 2020 to help economically in the overcoming of the socioeconomic crisis and to provide immediate financial support under criteria of flexibility in the funds allocated (European Commission, 2020). In this context, musicians with permanent employment have been more active in innovating and solving technological problems than self-employed and furloughed artists (Elstad et al., 2021), so job security may contribute to self-management capacity and entrepreneurship.

The health crisis has also harmed Latin American musicians, due to two aspects that are linked to deficient cultural policies available to support their professional work before the COVID-19 pandemic. On the one hand, they tend to be self-employed with insufficient social protection even before the COVID-19 pandemic (UNESCO Office Montevideo and Regional Bureau for Science in Latin America and the Caribbean et al., 2021). This has encouraged them to undertake cultural projects, giving an account on the capacity of self-management and entrepreneurship (Ponce de León, 2021). Moreover, several Latin American countries experienced socio-political instabilities from October 2019, which was applied to a curfew on live events (Angel-Alvarado et al., 2021). Hence, musicians learnt to implement new ingenious strategies for spreading the music before the health crisis, which was beneficial as this new environment served as artistic inspiration for resourcefulness. On the other hand, Latin American countries allocated funding for helping cultural and creative sectors (UNESCO Office Montevideo and Regional Bureau for Science in Latin America and the Caribbean et al., 2021). Still, there was a delay in the subsidies they received through a lack of funds and excessive bureaucracy for assisting beneficiaries (Isernia and Lamonica, 2021; Moguillansky, 2021). Therefore, the capacity to self-manage has been paramount for the sustainability of musicians.

At present, no research has been found that compares Western classical music performance activities or experiences by musicians located in European and Latin American contexts. In fact, most of the research in music performance has been dedicated to the first world countries, often ignoring the practices of developing countries. Given the descriptions of WHO (Sohrabi et al., 2020; WHO, 2020) regarding the vulnerability of these countries during the pandemic, it is relevant to focus on comparisons across cultures. In music, most of the cross-cultural empirical research focuses on comparing cohorts across Western countries, and the studied topics include perception (e.g., Stevens, 2012), cognition (e.g., Stevens, 2012; Jacoby et al., 2020), and affect (Susino and Schubert, 2020).

## Materials and Methods

Our study focuses on the challenges for classically trained musicians from Europe and Latin America during the peak of the COVID-19 pandemic in 2020—two cultural contexts with long traditions for classical music. We were particularly interested in how practice routines in these two regions changed as the numbers of concerts dramatically decreased due to cancelations. More specifically, we sought to understand whether certain forms of (harmonious/obsessive) passionate engagement with music practice predict the sustainment of music practice and whether the years of performing experience might be related to a change in practice routines.

### Research Questions

The question that guided our research was as: Do classical musicians in Europe and Latin America differ in their *engagement with music practice* before the pandemic and at the peak of the pandemic in 2020 according to:

a. their harmonious/obsessive passionate engagement with music,
b. their years of performing experience, and
c. their experiences due to socioeconomic and cultural contexts?

### Design

This study involved a cross-cultural, ex-post facto, and sequential explanatory mixed methods research design focused on classical musicians with active performing careers in Europe and Latin America. The study employed a survey to explore causal relationships between variables [i.e., (1) harmonious/obsessive passion; (2) years of performing experience; and (3) geographical context] in their music practice routines during the COVID-19 pandemic (i.e., average weekly accumulated practice of instrumental/vocal music).

The survey explored these aspects through the perceptions of the participating musicians during two pandemic stages: (1) immediately *before* the pandemic and (2) *at the peak* of the pandemic in 2020. Our analyses used quantitative and qualitative data from these two responses to describe patterns of change and help establish the direction and magnitude of the identified causal relationships.

### Participants

A total of 309 usable responses by classically trained, professionally active musicians were considered to perform the analyses. The sample included 150 males (48.5%) and 159 females (51.5%) whose age ranged from 18 to 75 years (*M* = 41.11; *SD* = 13.30). We received 206 responses (66.7%) from Europe and 103 responses (33.3%) from Latin America. A total of 155 different cities were represented within 30 European countries and 11 Latin American countries, as follows (in order of number of participants per country): Argentina (38), Finland (28), Spain (23), United Kingdom (20), Netherlands (20), Brazil (18), Chile (16), Peru (13), Germany (12), Bulgaria (11), Austria (9), France (7), Italy (7), Portugal (7), Switzerland (7), Estonia (6), Greece (6), Sweden (6), Belgium (4), Dominican Republic (4), Mexico (4), Norway (4), Uruguay (4), Cyprus (3), Hungary (3), Luxembourg (3), Poland (3), Romania (3), Czech Republic (2), Ecuador (2), Lithuania (2), Venezuela (2), Armenia (1), Costa Rica (1), Croatia (1), Georgia (1), Slovakia (1), Slovenia (1), Paraguay (1), and Ukraine (1).

The musicians displayed a variety of (multi-)professional roles within the classical music industry, such as instrumental/vocal soloists, chamber and/or orchestral musicians, ensemble and/or choir singers, or a combination of the above. From these, 20 musicians (6.47% of the participants) combined their professional performing activities with the final years of instrumental/vocal studies in higher music education institutions; something that is common for many students who are aiming to become professional musicians. The years of professional performing careers (performing experience) of the musicians ranged from 1 to 58 years (*M* = 19.85 years; *SD* = 12.58) and the distribution of participants per instrument(s)/voice(s) was as follows: bowed string instruments (102), wind instruments (53), keyboards (45), singers (44), brass instruments (32), plucked instruments (19), percussion (7), and a combination of different instruments/voices (7).

## Materials

### Adaption of the *Passion Scale for Music*

For this investigation, we adapted the previously validated *Passion Scale for Music* (PSM) developed by Bonneville-Roussy and Vallerand (2020) to examine musicians’ professional music practice routines before and during the COVID-19 pandemic. The original scale comprised 17 items related to musicians’ dualistic passion regarding their engagement with music and refined from the *Passion Scale* (PS) by Vallerand et al. (2003) originally developed to understand why people invest time and energy in certain activities and tested with large cohorts of people, including college students, football players, recreational cyclists, and casino gamblers. The PSM provides an averaged, continuous (and non-dichotomous) score of the passion profile displayed by different individuals involved in music making—understanding that people can be more or less passionate about any activity, and that there is a certain presence of both harmonious and obsessive passion in everyone.

The 17 items of the adapted PSM used in our survey were randomized and based on a seven-point Likert-type scale in the online survey as presented below. In our survey, the musicians were asked to indicate their level of agreement with each item before the COVID-19 pandemic, prompted by the following statement: *“We are interested in knowing more about how you felt about your musical involvement in the 6/12 months before the pandemic began. While thinking of your playing activity and practicing habits and using the scale below, please indicate your level of agreement with each item before the pandemic.”*

The adapted items corresponding to Harmonious Passion (HP hereinafter, six items), Obsessive Passion (OP hereinafter, six items) included in our survey were as:

- My practice was in harmony with the other activities in my life (HP).
- The new things that I discovered with my practice allowed me to appreciate it even more (HP).
- My practice reflected the qualities I like about myself (HP).
- My practice allowed me to live a variety of experiences (HP).
- My practice was well integrated in my life (HP).
- My practice was in harmony with other things that were part of me (HP).
- I had difficulties controlling my urge to do my practice (OP).
- I had almost an obsessive feeling for my practice (OP).
- My practice was the only thing that really gave me satisfaction in my life (OP).
- If I could, I would only do my practice (OP).
- My practice was so satisfying that I sometimes lost control over it (OP).
- I have the impression that my practice controlled me (OP).

In addition, we included the five-item criterion subscale that was part of the original PS (i.e., Vallerand et al., 2003) and the PSM (internal consistency *α* = 0.89 as reported by Bonneville-Roussy and Vallerand, 2020). The items included in this subscale respond to “the extent to which the activity is loved, valued, is a ‘passion’, as well as the time spent in the activity and integration of passion into the identity” (Bonneville-Roussy and Vallerand, 2020, p. 272); the adapted items of the subscale that we used for our research were as:

- I spent a lot of time doing my practice.
- I loved my practice.
- My practice was important for me.
- My practice was a passion for me.
- My practice was part of who I was.

### Years of Performing Experience Within the Classical Music Industry

International research on musicians’ career development taking the life span perspective as a theoretical starting point has acknowledged that early-career musicians tend to focus on different aspects than mid-career and late career musicians (e.g., López-Íñiguez and Bennett, 2020) and that the strategies to keep up their professional activity might fluctuate over time (e.g., Bennett and Hennekam, 2018). Furthermore, research specifically focused on the impact of the pandemic on musicians across the United Kingdom has identified that mid-career and late career musicians felt a “loss of a much-loved performing career, missing music making and colleagues, and anxiety about the future of the music profession” (Cohen and Ginsborg, 2021, p. 1), while the “challenges to their identity as a musician, the extent of their anxiety about finances, the extent of their emotional distress, attitudes toward practicing and engaging in collaborative music making, and confusion over future career plans” were different for each group. Considering these recent studies, we also asked the musicians about their years of professional work as performing musicians as a potential variable to understand the effects of the COVID-19 pandemic on their practicing routines.

### Average Weekly Practice and Experiences Regarding Practicing Routines Before and at the Peak of the COVID-19 Pandemic

Our survey also included questions regarding the practice habits/routines of the participating musicians immediately before the COVID-19 pandemic was officially announced in their respective countries of residence (approx. late 2019, and January–February 2020; BEFORE hereinafter), and around the time when the pandemic was at its peak in their place of residence in 2020 (approx. from April to December 2020; DURING hereinafter). Questions related to the musicians’ practice in this section of our survey gathered information on the average number of days spent practicing each week, and the typical number of hours practiced during each of these days (measured in an ordinal scale ranging from 0 to 9 points: from less than 30 min through to more than 8 h per day). Thus, as a criterion variable, we calculated the average Weekly Amount of Practice by multiplying the days per week by the number of daily minutes/hours spent practicing, which resulted in statistical differences when comparing the two stages of practice before and during the peak of the COVID-19 pandemic (Table 1).

**Table 1:** Average weekly amount practice by the participating musicians.

	Mean	*SD*	*T*	sig.
BEFORE the pandemic	22.86	13.44	7.55	0.000
DURING the pandemic	16.21	15.34		

In addition—as we wanted to understand how we could make sense of the musicians’ responses to the survey, as well as ensure the quality of the interpretation of results—we included two open-ended questions that asked the musicians to describe how they felt about their professional music practice before and at the peak of the COVID-19 pandemic. This provided a rich pool of genuine narratives from the musicians’ experiences regarding music practice during the periods studied. The open-ended questions were as: (1) *“Describe how you generally felt about practice before the pandemic”*; (2) *“Describe how you generally felt about practice at the peak of the pandemic.”*

### Procedure

Data were obtained from a public online survey that adapted the validated scales described above. The surveys were disseminated *via* Surveypal software in both English and Spanish. To access research participants in Europe and Latin America, we utilized a combination of probability randomized sampling and snowball sampling techniques. To do this, we contacted colleagues, institutions, and societies related to musicians’ professions, as well as professional orchestras, choirs, and ensembles seeking their help to distribute the survey invitations within their respective networks. We also arranged social media advertisements by targeting populations connected with the classical music industry. The survey was open from May 14 until June 30, 2021.

The approximate time needed to complete the survey was 15–20 min. Background questions related to the participants’ age, gender, country, and municipality of residence, details on where they worked, their professional performing profile, their years of professional experience as performing musicians, their employment status, and their main type of instrument/voice were also included in the survey. Finally, we asked the musicians about their professional concert activity, in terms of the typical number of live or streamed concerts they were involved in each month.

### Analyses of Data

Data analyses were performed using (1) SPSS 27 for the building of harmonious/obsessive profiles; (2) SPSS AMOS 20 for the path analyses (i.e., multigroup invariance) involving the studied variables; and (3) manual coding for the socio-cultural analysis of musicians’ narratives in the open-ended questions.

#### Assigning Harmonious/Obsessive Passion Profiles to the Participants

We employed a cluster analysis and a *χ*^2^ test to assign harmonious/obsessive passion profiles to the most representative participants of the passion continuum extremes. In order to determine the most extreme personal characteristic prototypes, in a first selective step, it was decided to separate the population that had scores above +1.5 SD of the variables either OP (*M* = 18.7242; *SD* = 7.25277) or HP (*M* = 31.5372; *SD* = 6.49546). Thus, only people with OP scores greater than or equal to 29.3 or HP scores greater than or equal to 41.2 were eligible for further analysis.

Thus, with this group of *n* = 31, a *K*-means cluster analysis was carried out in which a possible differentiation was sought between people who could have low levels of OP and high levels of HP, intermediate levels of OP and HP, and high levels of OP and low levels of HP. (Note: this process helped with the building of the models as described below, but also with the coding of qualitative data that was employed to elicit prototypical stories related to the quantitative results).

As indicated in Table 2, a significant grouping was found that separated both variables into three population groups with significant differences (OP: *F* = 71.869; *p* = 0.000/HP: *F* = 79.515; *p* = 0.000) between high, intermediate, and low levels of both variables, as expected theoretically. That is, people with high levels of OP report lower levels of HP and vice versa.

**Table 2:** Grouping of participants into different passion profiles.

		*N*	*M*	*SD*	*SE*	*F*	*p*
OP	LowOP_HighHP	6	11,3,333	4,45,720	1,81,965	71.869	0.000
	MediumOP_MediumHP	10	30,5,000	5,03874	1,59,339		
	HighOP_LowHP	15	33,8,000	2,75,681	71,181		
HP	LowOP_HighHP	6	42,0000	00000	00000	79.515	0.000
	MediumOP_MediumHP	10	41,7,000	67,495	21,344		
	HighOP_LowHP	15	33,7,333	2,46,306	63,596		

However, the *post hoc* analyses reveal that when analyzing the levels of OP, the main differences are referred to when comparing the means of the people included in the groups with low levels of OP and high levels of HP with the other two groups. In this way, Tukey’s HSD provides us with the existence of a homogeneous group of integrated people in which they report intermediate values of OP and HP and high values of OP and low values of HP (*p* = 0.195).

On the other hand, and with regard to the analysis of the groups belonging to the population based on the HP values, we see how the significant differences (*p* = 0.000) occur in the comparison between the people who present low levels of OP and high HP with the rest of the population, with no significant differences (*p* = 0.932) between the group of people with OP and intermediate HP and the group of people with low OP and high HP.

After this—and therefore assuming that the OP and HP variables can be dichotomized into high and low values—we find that depending on the continent there is no significant association in the OP variable (*χ^2^* = 0.149; *p* = 0.700), while however, there is a certain significant association between extreme, high, or low HP scores and the continent of origin of the participant (*χ^2^* = 4.210; *p* = 0.040). Regarding the low levels of HP, we find more people (*n* = 14) than expected (*n* = 11.6) of European origin and fewer people (*n* = 1) than expected (*n* = 3.4) of Latin American origin. In the case of people with high levels of HP, we see how those of European origin count (*n* = 10) less than expected (*n* = 12.4) while in the case of people of Latin American origin we count (*n* = 6) more than expected (*n* = 3.6).

#### Multigroup Invariance Analysis in Structural Equation Modeling

In general terms, we present below the models that yielded the best fit data in the different analyses of structural equations models. To do this, both before the pandemic and during the pandemic, different nested models were tested using invariance analysis. That is, a general unconstrained model was used as a starting point for both moments in which differences were assumed between the two population groups studied, and these differences were restricted until fully constrained models were achieved—where it is assumed that there are no population differences in the groups to be compared. In this sense, starting from unconstrained models, we saw how, to explain the different relationships between the variables, before the pandemic the best option was a totally constrained model (structural residuals constrained) in which we must assume that there are no differences between the two population groups. Notwithstanding, when talking about during the pandemic, it was necessary to assume that there were differences among both populations’ groups.

#### Interpreting the Quantitative Analyses Through the Lenses of Narrative Analysis

In this cross-cultural research, we understand that narratives and realities of individuals—in this study, related to their passionate engagement with music performing and their COVID-19-related experiences—are not exclusively personal, nor natural, but constructed as part of their social exposure to the culture and context where they live. In that sense, following Riessman (1993) as well as Coffey and Atkinson (1996), studying individual narratives as objects of enquiry is an important process to reveal socioeconomic and cultural values, practices, and conventions that people perform in their everyday social interaction. Thus, from the 31 most prototypical participants associated to harmonious and obsessive passions identified through the cluster analysis and the χ^2^ test described in section 4.5.1, we employed a psychosocial lens approach (i.e., Denicolo, 2003) to elicit the important elements that were perceived to be significant—through *post hoc* analyses—when musicians narrated their experiences before and during the peak of the pandemic when responding to the open-ended questions. We employed a manual thematic analysis that guided our short narrative interpretation of the quantitative analyses below in connection to the main critical frameworks of the study.

## Results

### Multigroup Invariance Analysis: Europe and Latin America Before COVID-19

According to the multigroup invariance analysis in structural equation modeling presented in the section “Multigroup Invariance Analysis in Structural Equation Modeling,” the best fitted model before the pandemic for both Europe and Latin America (CMIN/df = 0.822; *p* = 0.618; CFI = 1.0; RMSEA = 0.000) that accounts around the 19% of the variance of the Weekly Amount of Practice variable is the one in which we need to assume both groups are the same in terms of explanatory capacity of Weekly Amount of Practice and its inverse relation for both groups with the Years of Professional Experience (the more years the less practice: *β* = −0.13; *p* = 0.017), and the direct relation for both groups too between OP and practice (*β* = 0.15; *p* = 0.007) and HP with practice (*β* = 0.33; *p* = 0.000).

**Figure 1:**
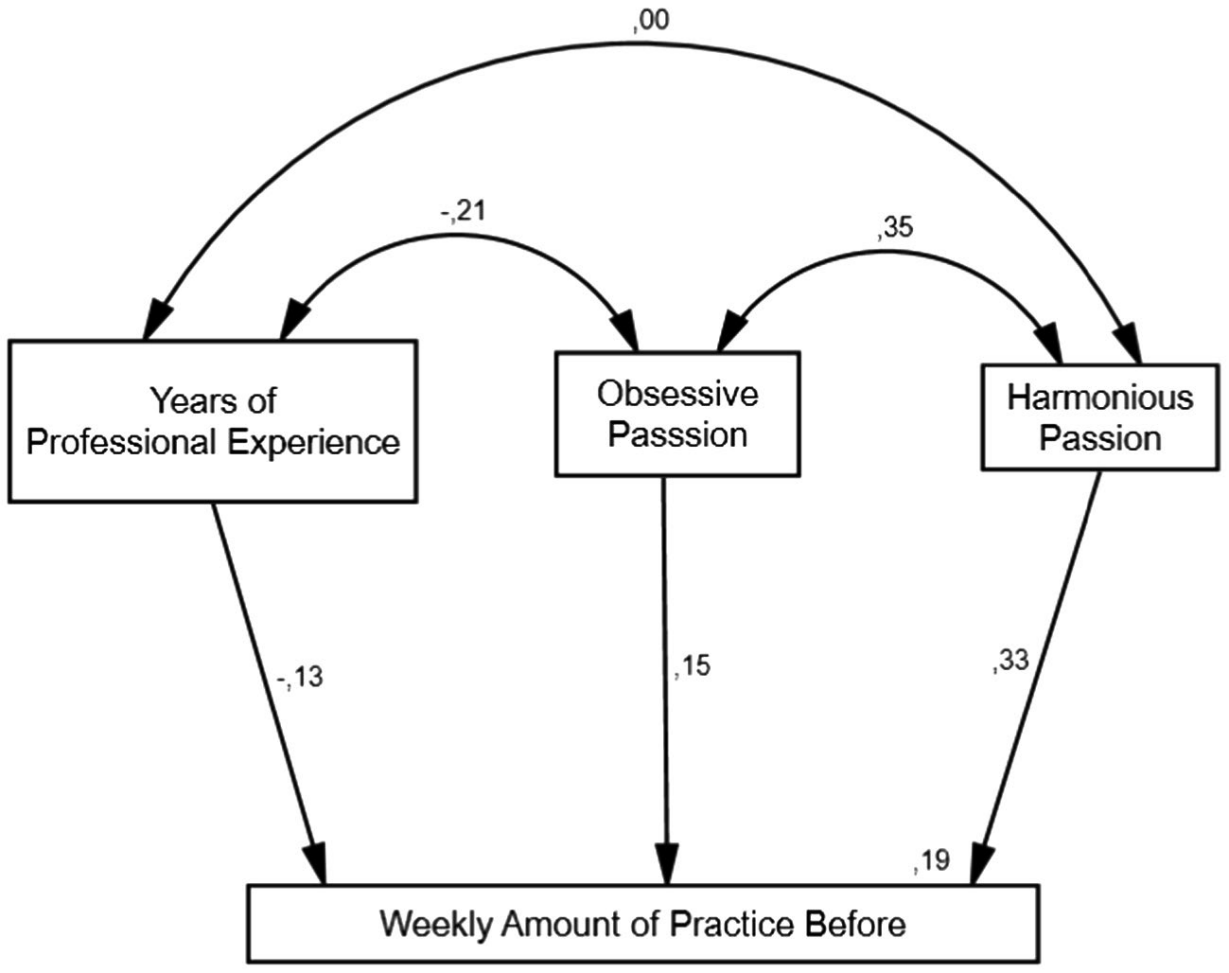
Model before the pandemic for Europe and Latin America.

Also noticeable is the inverse relation for both groups between the years and the OP (*r* = −0.21; *p* = 0.000), the direct relation for both groups between the OP and HP (*r* = 0.35; *p* = 0.000), and the non-significant relation for both groups between the years and HP (Figure 1).

### Narrative Analysis: Europe and Latin America Before COVID-19

The narrative analysis suggests that the practice of musicians before the pandemic could be explained through different profiles associated to a lesser/greater extent to harmonious and obsessive passion (understanding passion as an independent combination, i.e., a non-dichotomic variable), regardless of the geographical location of participants. In addition, the more years of performing experience participants possessed, the less practice they invested, and the less obsessive passion they displayed.

Regarding the musicians in the obsessive-focused passion group, they were goal-oriented as they “exclusively focused on the next concert.” Practicing time before the pandemic was understood as part of the “40-h job any professional has to do per week” and “never enough and always a means to an end for the next gig.” These musicians focused on technical mastery and muscle training to “stay in shape” and they practiced even if they had injuries—these aspects were considered by the participants as ways to maximize efficiency to “get the most out of [my practigint time].” Other professional and personal activities besides music practice disturbed them before the pandemic as they experienced being “in a hurry and juggling between several engagements both performing and teaching.”

The musicians belonging to the more harmonious-focused passion group, however, understood practice as integrated and consistent in their general life and in their profession before the pandemic: “I have worked hard for many years to integrate being a musician with being the person I am.” In this sense, the musicians had a positive and relaxed relationship with practice as they generally “prepared for concerts in a concentrated, happy, joyful, and efficient way” while doing “no more than was needed for professional purposes as, in my country, a professional musician depends on other activities to make a living, which is fine.” In addition, practice was understood as process-oriented, so participants planned what to do and why (e.g., “warming-up to have a good feeling” for their practice, using “discovery learning for fun”), and included practice outside the instrument whenever possible (e.g., “reading the scores when the music is technically easy”).

### Multigroup Invariance Analysis: Europe and Latin America During COVID-19

#### European Context

As discussed above, in the case of the analysis of the results of the structural equation models in charge of describing the relationships of the variables during the pandemic, the model that presented the best fit (CMIN/df = 1.012; *p* = 0.415; CFI = 0.999; RMSEA = 0.006) is the one in which we need to assume both groups are not the same and, therefore, that there are differences between the group of musicians from Europe and from Latin America.

Considering the overall model, this model explains 6% of the variance of the Weekly Amount of Practice variable for Europeans and 10% of the Weekly Amount of Practice for Latin American musicians. Also noticeable for both groups was a non-significant relationship between the Years of Professional Experience and the HP. In addition, there are no significant differences when comparing the models fit (*∆χ^2^* = 2.969; *df* = 5; *p* = 0.705), meaning both models (Before and During the pandemic) fit equally well for their sample.

Regarding the sample of Europeans and to explain around the 6% of the variance of Weekly Amount of Practice, we found a significant direct relationship between the Years of Professional Experience and Weekly Amount of Practice (*β* = 0.16; *p* = 0.024) as well as between the OP and Weekly Amount of Practice (*β* = 0.24; *p* = 0.000), but not between HP and the Weekly Amount of Practice. In terms of correlations, the variable Years of Professional Experience are inversely correlated with the OP (*r* = −0.25; *p* = 0.000) and OP and HP are directly correlated (*r* = 0.35; *p* = 0.000).

**Figure 2:**
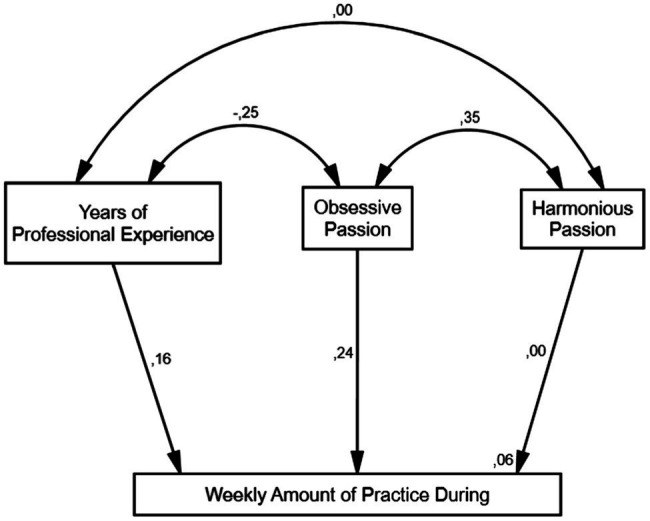
Model during the COVID-19 pandemic for Europe.

In light of these results, it is important to note that we tested the possibility that the size of the effect sample sizes in each context sample (6% in Europe and 10% in Latin America) could be influencing the differences in Weekly Amount of Practice. However, those differences were non-significant (*F*-Before = 3.150; *p* = 0.077; Levene = 0.790/*F*-During = 1.100; *p* = 0.295; Levene = 0.427; Figure 2).

#### Latin American Context

For musicians in Latin American, the only direct relationship to explain Weekly Amount of Practice was the relation between HP and Weekly Amount of Practice (*β* = 0.31; *p* = 0.000). Therefore, there is no relation between: Years of Professional Experience and Weekly Amount of Practice, OP and Weekly Amount of Practice, or Years of Professional Experience and OP.

**Figure 3:**
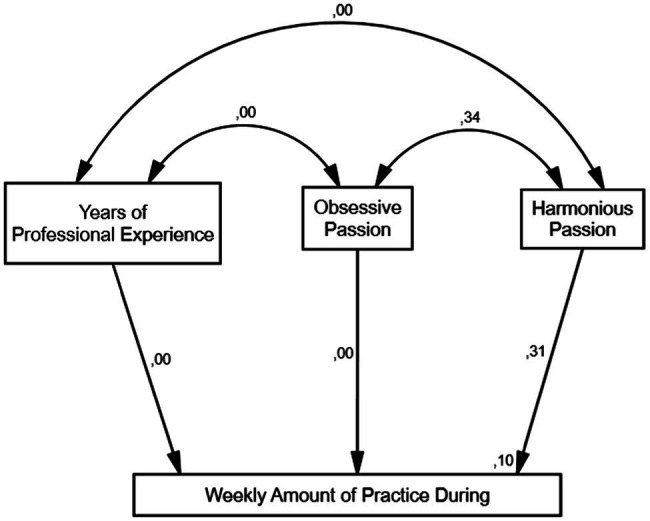
Model during the COVID-19 pandemic for Latin America.

Analysis of the correlations between variables shows that there was a significant direct correlation between OP and HP (*r* = 0.34; *p* = 0.000); nevertheless, its indirect effect over the Weekly Amount of Practice was not significant. Considering all the relations describe above, the model explains around 10% of the variance of the Latin Americans’ Weekly Amount of Practice (Figure 3).

### Narrative Analysis: Europe and Latin America During COVID-19

As shown in the multigroup invariance analysis for Europe and Latin America during the peak of the pandemic, changes occurred according to practice and the geographical location mattered since obsessive passion was not the driver/trigger for musicians’ practice in Latin America in comparison with that of those based in Europe. In other words, harmonious passion explained the practice in musicians located in Latin America, whereas obsessive passion and more years of performing experience explained the practice in people based in European countries. In addition, there were musicians located in Europe who displayed a more harmonious passion profile and remained in a sort of “stand-by” position, in which they sought to wait out the pandemic.

From the narrative analysis, musicians in Europe displayed a more obsessive type of passion, for whom other professional and personal activities [such as other (non-)music-related jobs or studies] disturbed their practice across the pandemic as “all activities outside studying my instrument took more time and energy from me once they became part of the online format.” These musicians felt the pandemic had a certain positive impact on their lives because they “finally had time to practice more,” although at the same time, they felt helpless as practice “is never enough and unfruitful even if I spend many hours” just for the “necessary muscle training.” Practice was seen as a “personal space” (coping strategy) to keep mentally and emotionally away from the pandemic situation “while waiting for the situation to improve and become better” (in the sense of the restrictions being lifted and the pandemic disappearing). This helped them to immerse themselves on repertoire they might not perform, for instance by spending “10 months learning a piece that I may never perform. Now that’s cool.” In general terms, these musicians felt “unfocused, worried, confused, and depressed” as well as “aimless, unsure about what to aim for and where the next goal was.” Some of these musicians held a full-time post as a music teacher or orchestral/choir musician, so they mentioned that the pandemic did not “bring a huge change” to their life, and that practice was more “functional than aspirational.” However, some musicians with less stable jobs were worried about the music market being rather “hard to access; I would probably have a nervous break-down if I was just a musician,” or about the “concert and festival organizers finding me interesting enough anymore.”

In contrast, musicians in Latin American countries who belonged to the more harmonious-focused passion group kept their “practice routines consistent before and during the pandemic,” partly due to multifaceted tasks such as “videocalls with friends to play music together” or “digital concerts that I was already used to doing before the pandemic.” These musicians connected practice to a sort of existentialism in connection to their economic fragility as self-employed, since “all of us who practice music as a way of life have faced interminable situations that have led us to reflect on the purpose of a musician and the arts” and “we aren’t paid enough in general, though this was an additional outlet to stay active.” They mentioned that the peak of the pandemic was a time that allowed them to have “time for resting, which is also important for human beings.” In that line, not having live concerts during this period gave these musicians time for other things considered important in their life besides music practice: “I am delighted to have a reason for working on other non-musical and personal aspects of my life that pre-pandemic business had forced me to overlook”; “I did not play the violin for almost 3 months, but I did not feel bad about not playing. I knew it would be harder later, but in the beginning, resting was good for me.” Practice was rewarding and strongly linked to personal enjoyment and a meaningful career, for example, by “adding non-orchestral pieces to enjoy now when I get a chance for it,” “rediscovering the joy of playing for myself,” or establishing “a renewed connection with my instrument through artistic and career meaning-seeking.” Practice was also connected by these musicians with maintaining mental health, as at the peak of the pandemic “practicing was a very important tool to manage the anxiety of the moment,” being “more focused on the health of the musician, not so much on the development of technical or interpretive skills,” and “feeling safe and calm, finding respite in it.”

## Discussion and Conclusion

In this study, we were interested in understanding potential differences between professional performing classical musicians who were located in Europe and Latin America, and in particular their engagement with music practice immediately before and at the peak of the COVID-19 pandemic in 2020. For this, we focused on three main variables that were considered to have a potential impact on that engagement: (1) the type of passionate engagement with music, (2) their years of performing experience, and (3) their experiences according to socioeconomic and cultural contexts. The results of this study suggest a number of findings.

First, distinguishing between musicians who display a harmonious vs. obsessive passion for music allowed us to focus on the influence of intrinsic vs. extrinsic factors, which govern music involvement at the professional level. Using this framework, we observed subtle differences in the profiles of musicians who displayed more harmonious or more obsessive passion for music. However, it should be noted that harmonious and obsessive passions are not understood as a dichotomic variable, but as an independent combination found across participants in different degrees (low, medium, and high presence for each type). In general, there were no differences according to the geographical context in the levels of practice these musicians displayed before the pandemic, whereas we found differences in the way we can explain the participants’ practice at the peak of the pandemic depending on the continent on which they were located.

Before the pandemic, the different mixtures of these two passions explained the weekly amount of practice of musicians, regardless of their geographical location. Musicians with higher obsessive passion were more likely to focus on concert engagements and the necessary mastery of the craft required to keep up with professional duties, even if they had physical injuries. They were also disturbed by other multifaceted tasks besides their music job(s) (in line with Barlow and Morrow, 2021). In comparison, more harmonious passion-oriented musicians were more inclined to focus on the practicing process as means of both being reflective practitioners, but also having a positive relationship with music, which they understood as an important part of their lives and identities (in line with Vallerand, 2008). In relation to the years of performing experience, this variable impacted the results before the pandemic so that the more years of professional experience a musician possessed, the less practice that was invested. During this period, this variable was not connected to the geographical location, but to musicians displaying a more obsessive passion.

Thus, during the peak of the pandemic, change occurred and the geographical context (when dividing Europe from Latin America) mattered (Moreno, 2022), as the driver/trigger or impediment/obstacle for weekly amount of practice in people could be explained in terms of their distinct passionate profiles. In fact, harmonious passion explained a more sustained and higher weekly amount of practice in Latin American people due to their capacity for self-management and entrepreneurship (Ponce de León, 2021), whereas obsessive passion explained the practice by people in Europe due to the fragility of the cultural and creative sectors, which was revealed suddenly (Dümcke, 2021; Baltà, 2022). However, we also found people with more harmonious passion profiles in Europe who remained in a “stand-by” position during this period, hanging-on to survive. At the peak of the pandemic, however, the years of performing experience impacted the results by explaining differences between European and Latin American contexts. Thus, the engagement with music practice was related to more years of professional experience in musicians located in Europe and who were representative of the higher obsessive passion profile. In contrast, the practice of musicians in Latin America can be explained only by their harmonious passion.

According to the cultural politics affecting musicians in Europe and Latin America, we identified subtle differences in the testimonies of these musicians. For instance, musicians located in Latin America described having less economic safety and even an existential connection to a music profession that often does not generate enough revenue for them. However, they kept practicing and displayed harmonious passion at the peak of the pandemic. As discussed earlier, there has been a clear division of available governmental resources for cultural industries in these contexts. Following the study of Elstad et al. (2021), this could be understood as an effect of cultural politics, as the weakness of the cultural trade in particularly Latin American countries forces musicians to be self-employed and embrace their economic fragility (UNESCO, 2022). In that way, it could be argued that these musicians’ “positive” response (in terms of practice and passion) to the pandemic is a type of political stance that serves to answer the bad public management in cultural terms (Isernia and Lamonica, 2021; Moguillansky, 2021). Furthermore, the lack of resources in Latin American countries have forced musicians to manage their careers across long periods of time, much before the pandemic (UNESCO Office Montevideo and Regional Bureau for Science in Latin America and the Caribbean et al., 2021), whereas in Europe musicians generally have chances to obtain scholarships, subsidies, or contracts and professional possibilities of all sorts, in addition to a growing focus on entrepreneurial education (e.g., European Commission, n.d.).

However, we need to be cautious with these results, as, for example, in the United Kingdom, musicians who have been described as being “engaged in enjoyable, busy, successful, portfolio careers [before the pandemic, were]… unable to earn a living carrying out their everyday work of performing music, and their future working lives [were] surrounded by great uncertainty” (Cohen and Ginsborg, 2021, p. 1). Thus, further studies should investigate the different forms of employment of these musicians and how these relate to and impact on their practice and rehearsing routines during times of crises such as was observed during the COVID-19 pandemic. Furthermore, we suggest that the preparations of professional musicians for the workforce should attend not only to entrepreneurship models connected to neoliberal agendas, but also to the ongoing fragility and vulnerability experienced in (and out of) times of crises (in line with Cohen and Ginsborg, 2021), so that they could better respond to unexpected events such as COVID-19.

## Data Availability Statement

The datasets presented in this article are not readily available because data are not available in any open data repository. A selection of the pseudonymized datasets generated for this study is available on request from the corresponding author. Requests to access the datasets should be directed to guadalupe.lopez.iniguez@uniarts.fi.

## Ethics Statement

The study’s ethical acceptability was reviewed by the Research Ethics Committee at the University of the Arts Helsinki on 19.4.2021. All participants received sufficient information about the research study and its privacy terms and provided their written informed consent to participate in this study.

## Author Contributions

GL-Í conceived the original idea of the research, applied for the ethical review of the research, prepared, published, and spread the survey internationally, and collected, screened, and translated the data from the Spanish speaking participants into English. GL-Í and GM undertook the initial designing of the study and drafted the first version of the manuscript. GL-Í, GM, and RA-A formulated the conceptual ideas adapted in the manuscript. GL-Í and FZ, respectively, performed the qualitative and quantitative analyses with feedback from GM and RA-A. GL-Í, GM, FZ, and RA-A provided critical feedback at all phases, discussed, and contributed to the interpretation of the results and writing of the manuscript. All authors contributed to the article and approved the submitted version.

## Funding

This work was funded by the Jenny and Antti Wihuri Foundation as part of the first author’s research project *Expanding reflexivity of professional education in music in times of crises*. We also acknowledge the financial support of the Doctoral Education and Research Steering Group (TTOR) at the University of the Arts Helsinki for the coverage of the article Open Access processing charges in this journal.

## Conflict of Interest

The authors declare that the research was conducted in the absence of any commercial or financial relationships that could be construed as a potential conflict of interest.

## Publisher’s Note

All claims expressed in this article are solely those of the authors and do not necessarily represent those of their affiliated organizations, or those of the publisher, the editors and the reviewers. Any product that may be evaluated in this article, or claim that may be made by its manufacturer, is not guaranteed or endorsed by the publisher.
